# Feasibility of restricting e-cigarettes to prescription only for smoking cessation

**DOI:** 10.1186/s12931-024-02792-4

**Published:** 2024-05-09

**Authors:** Katya Peri, Mark J. Eisenberg

**Affiliations:** 1 Lady Davis Institute for Medical Research, Jewish General Hospital, Montreal, QC Canada; 2 Departments of Medicine and of Epidemiology, Biostatistics and Occupational Health, McGill University, Montreal, QC Canada; 3 Divisions of Cardiology and Clinical Epidemiology, Jewish General Hospital, McGill University, 3755 C?te Ste-Catherine Road, Suite H-421.1, Montreal, QC H3T 1E2 Canada

**Keywords:** Vaping, e-cigarettes, Regulation, Smoking cessation, Youth

## Abstract

E-cigarette use among youth in Canada has risen to epidemic proportions. E-cigarettes are also moderately useful smoking cessations aids. Restricting e-cigarettes to prescription only smoking cessation aids could help limit youth’s access to them while keeping them available as therapies for patients who smoke conventional cigarettes. In Canada, drugs or devices must be approved by regulatory bodies such as Health Canada in order to become licensed prescription medications. A similar situation is underway in Australia, where e-cigarettes have been restricted to prescription only. This commentary explores the feasibility of a similar regulation for e-cigarettes in Canada as prescription smoking cessation aids.

## Introduction

E-cigarettes (ECs) are handheld electronic devices that heat liquids containing nicotine to administer aerosols to the user [1]. E-cigarettes deliver nicotine to the brain more rapidly than other nicotine replacement therapies (NRTs) and mimic the sensorimotor and behavioural aspects of smoking a conventional cigarette [2]. This has made them popular among smokers. It has also provided arguments for their use as a smoking cessation aid [2]. In addition, the use of e-cigarettes among youth has risen to epidemic proportions [3]. A concern is that there appears to be a correlation between EC use in youth and subsequent tobacco smoking, which leads to harmful health effects on children and their health [4–8]. Whether ECs act as a gateway to tobacco smoking remains to be clarified, however, the correlation between these behaviours warrants attention. To mitigate these effects, legislation should be enacted and enforced on the sales of e-cigarettes. In addition, even more restrictions could be enacted to ensure that e-cigarettes are only available via prescription as medically licensed smoking cessation aids. Restricting their distribution to prescription only will potentially provide a way to limit the use of e-cigarettes among youth, while providing access to smokers who are trying to quit smoking traditional cigarettes.

## ECs as smoking cessation devices

A 2022 Cochrane review revealed that quit rates were superior in participants in the nicotine EC arm compared to the NRT arm (RR 1.63, 95% CI 1.30 to 2.04; I2 = 10%; 6 studies, 2378 participants) [9]. There is also evidence that ECs without nicotine improve quit rates compared to NRTs (RR 1.94, 95% CI 1.21 to 3.13; I2 = 0%; 5 studies, 1447 participants). A recent meta-analysis revealed that when used daily, ECs encouraged successful quitting (OR = 1.529; 95% CI = 1.158, 2.019; *P* = .005) [10]. These results suggest that if ECs are used daily, they can be effective smoking cessation therapies. This manner of e-cigarette use can potentially be implemented if ECs are recognized as prescription only smoking cessation aids.

While a Cochrane review has revealed that the current body of literature is suggestive that ECs could be useful smoking cessation aids, the report also calls for additional research to form a concrete conclusion [9]. Additional research is required to confidently assess the efficacy and safety of ECs for smoking cessation. By performing knowledge syntheses as the number of clinical trials increases, researchers will be able to have a clearer picture of the benefits and harms of ECs. 

## ECs and youth

Despite their potential as a smoking cessation aid, e-cigarettes pose a risk to the health of youth. Among Canadian youth aged 15–17 years old, 21.3% reported e-cigarette use in 2022 [11]. Approximately 89% of youth aged 15–19 years old who reported using e-cigarettes within the last 30 days used e-liquid with nicotine [11]. Developmental deficits in memory and executive function as well as cognitive impairments with memory have been associated with early nicotine exposure [12]. Furthermore, youth who vape are also more likely to experiment with tobacco cigarettes compared to youth who do not vape. In a 2018 study published in JAMA, researchers found that youth who used e-cigarettes were 20.5% more likely to subsequently use a tobacco cigarette [13]. In Canada, 66% of youth who smoke tobacco cigarettes reported first experimenting with e-cigarettes [11]. Current interventions to prevent youth from using e-cigarettes include flavour bans, age restrictions, warning labels, e-cigarette taxes and mass media awareness campaigns [14]. Despite attempts at mitigating youth vaping by restricting sales to minors and enforcing these bans, the number of children and adolescents who vape is still at large proportions [15]. Restricting e-cigarettes to prescription only could potentially help decrease the number of children vaping and smoking.

## EC regulation in Canada

Various legislative initiatives provide a legal framework for EC products in Canada. These initiatives are monitored for compliance and inspection by Health Canada. Regulation is primarily done under the Tobacco and Vaping Products Act (TVPA), however, the Canada Consumer Product Safety Act, the Food and Drug Act and the Non-smokers’ Health Act also make up the legal framework of EC regulation. However, it appears that the sales restrictions to minors may not be strictly enforced. To be considered licensed prescription medications, e-cigarettes must be approved as medical therapies [16]. Because the current body of evidence surrounding the long-term effects of e-cigarettes on patients’ health remains inconclusive, it is illegal to promote e-cigarettes as products that provide health benefits to their users [17]. In fact, EC packaging is required by law to include the following warnings “Vaping products contain nicotine, a highly addictive chemical” and “Vaping products release chemicals that may harm your health” [18]. Marketing ECs for smoking cessation or suggesting they are safer alternatives to tobacco cigarettes is therefore not allowed.

## ECs as prescription smoking cessation aids

For ECs to be restricted as prescription smoking cessation aids in Canada, the health benefits of ECs as smoking cessation aids must be clearly shown to outweigh the risks. For this to happen, large-scale, trails that evaluate all varieties of potential ECs on the market must be conducted. These studies must conclude that ECs are beneficial as smoking cessation aids. Health Canada must then approve ECs for smoking cessation. This process involves gathering clinical evidence and determining that the therapeutic benefits of ECs as smoking cessation aids outweigh the risks. Amendments to the access, promotion and labelling sections of the TVPA, which “regulates the manufacture, sale, labelling and promotion of tobacco products and vaping products sold in Canada” would be required, as well as amendments to the Food and Drug Act. Specifications for the advertisement and promotion of ECs as drugs is required in the Food and Drug Act. Importantly, new laws or Acts of Parliament would be necessary to ban the commercial sale of ECs. To license their products as therapeutic devices, EC companies would have to submit the appropriate evidence proving their product falls under the category of smoking cessation aid as per the federal government’s new stipulations. Once approved, these ECs would be available in pharmacies only as prescription medications.

E-cigarettes present a unique dilemma: while they offer potential benefits distinct from traditional cigarettes, restrictions designed to manage their risks can inadvertently hinder access for those who stand to gain the most. Balancing these factors within regulatory frameworks is crucial. For instance, categorizing e-cigarettes as prescription devices could provide a middle ground, allowing for less restrictive regulations while still ensuring controlled access. This approach acknowledges the potential benefits of e-cigarettes while addressing concerns about their widespread availability and use.

## EC use in Australia

One country Canada can potentially model its future EC regulatory framework from is Australia. In Australia, nicotine e-cigarettes are prescribed medicines and are only accessible from a doctor [19]. The Therapeutic Goods Administration (TGA) classifies nicotine used for non-therapeutic purposes as a dangerous poison (schedule 7 substance), restricting it to prescription only [20]. To ensure that the minimum quality and safety standards are met for nicotine e-cigarettes, the TGA has issued the *Therapeutic Goods (Standard for Nicotine Vaping Products (TGO 110) Order 2021* [21]. Prescribers can access unapproved drugs via three pathways: the special access scheme, the personal importation scheme and the authorized prescriber scheme [22]. The guidelines of the Royal Australian College of General Practitioners (RACGP) state that nicotine containing e-cigarettes should not be used as a first line treatment for smoking cessation. They are only recommended for patients aged 18 years or older if conventional pharmacotherapies and interventions fail and the patient remains motivated to quit smoking.

## EC use in the UK

Since EC restriction to prescription is unprecedented in North America, legislative and regulatory actions undertaken by other countries can serve as a model to follow. Canada can turn to its Commonwealth neighbour, England, for inspiration on how to implement EC restrictions to prescription only. In the UK, ECs have been endorsed by the UK Royal College of Physicians as acceptable smoking cessation aids. The National Institute of Health and Care Excellence has added ECs to the recommended list of smoking cessation therapies, mostly due to ECs being a safer alternative to tobacco smoking. A pilot project conducted in Greater Manchester involved the introduction of free ECs for smoking cessation in pharmacies. At the end of the study, 25% of smokers stopped using ECs and tobacco cigarettes after four weeks while 61% of those still smoking reduced their consumption by five cigarettes a day. In addition, the attitudes of both clients and service providers were mainly positive. This project is an example of the steps the UK is taking towards becoming smoke-free by 2030.

Although the UK does not have a prescription scheme like Australia does, the Medicines and Healthcare Products Regulatory Agency (MHRA) provides support and guidelines for companies wishing to license their ECs as medical devices. An application package including safety and quality evidence for both the medicinal product (e-liquid) and the device (pod/cigarette) must be approved in addition to non-clinical safety measures. Specifications standardizing the testing of the product must be met and statistical measurements of clinical variables (i.e. blood nicotine concentration) need to be approved. In addition, details regarding the manufacturing, packaging, processing and importation of these products are strictly monitored. With appropriate modifications, a similar regulatory framework could potentially be adopted in Canada. Restricting ECs to prescription only is feasible with the appropriate legislative and regulatory actions in place.

## Feasibility of restricting ECs to prescription only in Canada

The feasibility of a Canadian model similar to Australia’s is relatively low and many barriers to their restriction as medical devices exist. The main barrier is that there is a lack of consensus as to the efficacy and safety of ECs. Compared to the NRTs that are currently available, ECs promote moderate rates of smoking cessation. However, Health Canada will not endorse the medical use of a product if their safety outcomes are unknown or significantly harmful to public health. Studies evaluating the safety of ECs are therefore required, in addition to those evaluating their efficacy as smoking cessation aids. Another significant barrier is that without proper evidence, the amendments to legislative acts regarding EC regulation are less feasible. Resistance at the level of primary care practitioners is also a barrier to making ECs prescription only. Studies have shown that physicians are reluctant to discuss the benefits and risks of ECs with their patients and are not convinced of their efficacy for smoking cessation or their safety [23]. Currently, EC regulatory documents, like the TGA, do not provide guidelines for physicians regarding dose, duration and type of EC. In addition to resistance at the clinical level, EC companies are not likely to pay for the extensive testing and certification process required to make their device a prescription medication. Restricting ECs to prescription only could at once provide the tobacco-smoking population with a useful cessation aid and curb the use of ECs in youth. In addition, the crux of the matter is not solely the consensus regarding effectiveness but rather the expenses, time investment, and intricacies involved in assembling product documentation and conducting clinical trials. Considering recent studies, it seems plausible for companies to navigate current regulations successfully. However, the challenge arises from the lack of motivation to undergo this costly process when there’s a simpler alternative: direct sales to consumers. Despite these significant benefits to public health, there exist significant barriers that would have to be overcome to implement this initiative.

## Conclusion

As it stands, restricting ECs to prescription only is not currently feasible in Canada, mainly due to the lack of consensus surrounding the efficacy and safety of ECs as smoking cessation therapies. Despite this uncertainty, the research clearly shows ECs are a danger to youth. If ECs are eventually deemed safe and efficacious for smoking cessation, a major legislative overhaul of EC regulation would be needed to first suspend them for commercial use and limit them to prescription only. Limiting ECs to prescription only is a vital and delicate step that will require motivation and collaboration between governmental regulatory bodies, healthcare organizations and EC companies. Furthermore, it is unlikely that EC companies will be willing to financially support the testing and licensing procedures required to approve their products as medical devices. These procedures, although impractical, are not impossible and must be carefully considered with respect to existing models if the benefits to public health might outweigh the risks when it comes to prescription ECs. A regulatory framework similar to that of Australia could potentially benefit Canada in the fight against tobacco smoking.

## Data Availability

Not applicable.
